# Diagnosis and minimally invasive treatment of type III choledochal cysts

**DOI:** 10.1186/s12893-022-01713-w

**Published:** 2022-07-14

**Authors:** Guang-zhen Wu, Qing-yuan Wu, Zhi-hao Zhao, Meng Wang

**Affiliations:** Department of Hepatobiliary and Pancreatic Surgery, The First Hospital of Jilin University, 71 Xinmin Street, Changchun City, Jilin Province China

**Keywords:** Type III choledochal cysts, Minimally invasive treatment, Laparoscopic hepaticojejunostomy, Choledochocele, Case report

## Abstract

**Background:**

Type III choledochal cysts (CCs) are the rarest and least well-characterized of the five variants of biliary cysts. Only a few relevant studies have been conducted and a gold standard treatment for type III CCs is still lacking because of their scarcity. An unusual case of type III CC with choledochocele at the end of the distal common bile duct (CBD) with no bulging of the duodenum is presented here.

**Case presentation:**

A 61-year-old woman presented to our department with repeated upper abdominal pain for one year. Laboratory examination revealed abnormal liver function and a slight increase in the white blood cell (WBC) count and proportion of neutrophils. In an MRCP of the patient, the distal CBD was found to have a cystic structure indicative of a type III CC. Endoscopic retrograde cholangiopancreatograpy (ERCP) revealed cystic findings compatible with Todani type III CCs. However, duodenoscopy did not show a bulge in the duodenum so laparoscopic cholecystectomy and Roux-en-Y hepaticojejunostomy were performed. In her 6-month follow-up, the patient reported that recovery from the operation was uneventful.

**Conclusions:**

ERCP has become the gold standard for diagnosing type III CCs. Type III CCs can be treated minimally invasively with laparoscopic cholecystectomy and Roux-en-Y hepaticojejunostomy.

## Background

Biliary cysts, or choledochal cysts (CCs), are premalignant cystic transformations that require surgical intervention. CCs are a rare cystic dilation of the biliary tract, which were classified into five variants by Todani and colleagues in 1977. Type III CCs, also known as choledochoceles, are the rarest of all the CC variants, occurring in only 1.4–4.5% of large case series [1].

In spite of their rarity, CCs are important to study since they must be recognized and treated according to their symptoms to prevent biliary malignancies [2, 3]. Type III CCs diagnosis is primarily based on the anatomical location and characteristics of the cysts. Type I, II and III cysts are all extrahepatic bile duct cysts, and type III cysts can be easily confused with those of type I and II. There are three types of type I cysts, namely 1A–AC. Cystic dilation of the common bile duct (CBD) is the characteristic of type I cysts. Cystic dilation of the extrahepatic biliary tree is a characteristic feature of Type IA, as is an anomalous pancreaticobiliary junction (APBJ). With type IB, the extrahepatic biliary tree is segmentally dilated without APBJ, while with type IC, the extrahepatic biliary tree becomes diffuse fusiform dilation with APBJ. In type II cysts, the extrahepatic duct has discrete diverticula connected by a narrow stalk to the CBD. A type III cyst usually presents as a cystic dilation of distal CBD that extends into the duodenum. The most common clinical symptom of type III cysts is abdominal pain [4]. A standard method of diagnosis and treatment of this anomaly has not been developed due to its rarity. Laparoscopic choledochojejunostomy is the foremost surgical approach used to treat type III CCs. This case study describes a type of isolated dilatation at the end of the CBD in an elderly woman diagnosed with type III CCs using intraoperative choledochoscopy.

## Case presentation

A 61-year-old woman presented to our department with repeated upper abdominal pain for one year without vomiting, jaundice, chills, or fever. The onset of pain was initially every 2–3 weeks but had recently increased to every 1–2 weeks. During this period, she was diagnosed and treated for chronic nonatrophic gastritis at the gastroenterology clinic several times, but her pain was not relieved. No special procedures were performed prior to admission, except for abdominal ultrasonography (USG) at a local hospital. The patient did not have any history of addiction to nicotine or alcohol, weight loss, hypertension, cancer in the family, diabetes, nor did she have a previous surgery. No abnormalities were found during a physical examination. The following laboratory tests were conducted after admission, including electrolyte levels, routine blood tests, tumor marker levels and liver and kidney function. An abnormal WBC value was detected of 11.78 × 10^12^/L [reference range (RR) is 4.0–9.0 × 10^12^/L], neutrophilic granulocyte percentage was 0.77 (RR, 0.5–0.7). Abnormal liver function indicators were as follows: alanine aminotransferase (ALT) concentration of 353.03 IU/L (RR, 5–40 IU/L), aspartate aminotransferase (AST) concentration of 55.48 IU/L (RR, 8–40 IU/L), γ-Glutamyl-converting enzyme (γ-GT) concentration of 394.48 IU/L (RR, 8–57 IU/L), leucine aminopeptidase concentration of 123.77 IU/L (RR, 10–40 IU/L), glutamate dehydrogenase concentration of 56.31 IU/L (RR, 2–10 IU/L), and alkaline phosphatase concentration of 230.54 IU/L (RR, 30–120 IU/L). A measured bilirubin level, amylase level, lipase level, or tumor marker did not show any abnormalities. The immune globulin G4 level was also within normal ranges.

**Fig. 1:**
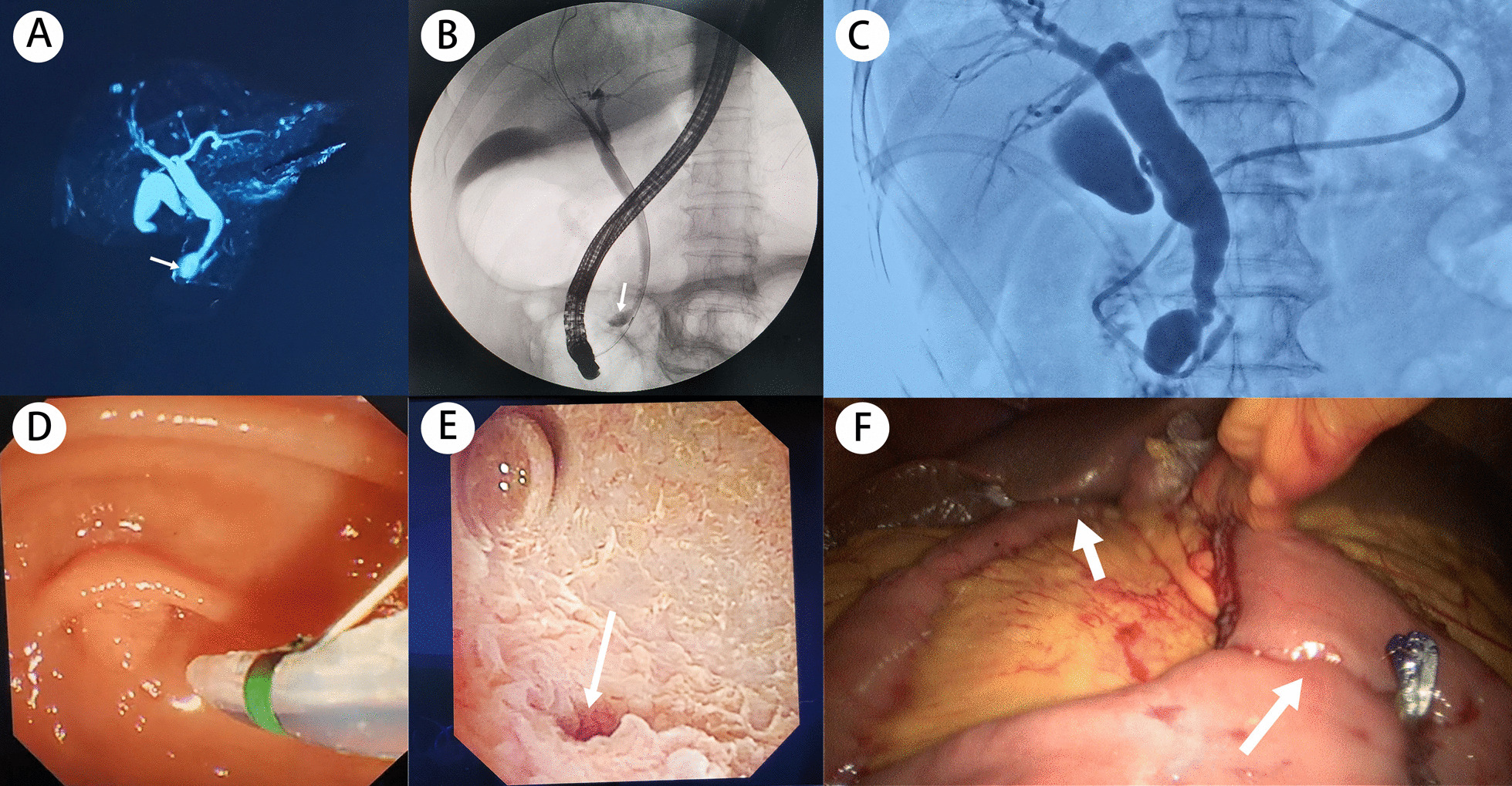
Preoperative and intraoperative findings. **A** MRCP showed cystic dilatation of the distal common bile duct (arrow). **B** ERCP showed cystic dilatation of the distal common bile duct (arrow). **C** ERCP showed an anomalous pancreaticobiliary duct junction. **D** Duodenoscopy showed that the duodenal papilla was flat and granular with no protrusions. **E** Choledochoscopy showed the opening of the cyst (arrow). **F** Laparoscopic hepaticojejunostomy was used to treat the type III cysts

The patient completed relevant imaging examinations at our hospital. The deep location of the distal CCs and intestinal gas accumulation prevented USG from detecting the cyst at the end of the CBD but showed that the diameter of the CBD in the upper pancreas was slightly widened, with the widest part measuring 10 mm. The abdominal USG found that the pancreas had a normal shape with a smooth contour, homogeneous echogenicity, and no dilatation of the pancreatic duct. Abdominal CT and MRCP were arranged for the patient. MRCP showed a cystic structure (15 × 15 mm) in the distal CBD, suggestive of type III CCs (Fig. 1A) but failed to show the pancreaticobiliary duct junction union. The patient consented to undergo endoscopic retrograde cholangiopancreatography (ERCP) which revealed cystic findings compatible with Todani type III CCs (Fig. 1B) and an anomalous pancreaticobiliary duct junction (APBDJ) (Fig. 1C). During ERCP, the concentration of amylase in the bile at the end of CBD was 90,349 U/L. We determined the definitive diagnosis of type III CCs based on the patient’s medical history, signs, and symptoms, and auxiliary examination results. Duodenoscopy did not recognize the bulge but identified a flat, granular opening of the duodenal nipple in the duodenum (Fig. 1D). As a result, sphincterotomy was not appropriate and minimally invasive surgery was ordered for the patient.

After ERCP, serum amylase and urine amylase levels were both slightly elevated (161.7 U/L and 988 U/L, respectively) but abdominal USG showed no signs of pancreatitis. Laparoscopic surgery was performed when all parameters returned to a normal range and was used in combination with choledochoscopy to detect the lesion. The spherical cyst cavity had a honeycomb appearance under the choledochoscope, and the opening of the bile duct merged into the pancreatic duct (Fig. 1E). These observations were consistent with the ERCP results. Thus, laparoscopic cholecystectomy and Roux-en-Y hepaticojejunostomy were performed, and the proximal CBD was clamped to reduce the stimulation of bile to the cystic wall and limit the bile duct pressure (Fig. 1F). The patient’s postoperative serum amylase, urine amylase, and serum lipase parameters were within normal limits, and AST, ALT, γ-GT, and other indicators returned to a normal range within 5 days after surgery. Abdominal USG showed no signs of pancreatitis or abdominal effusion. Following the surgery, the patient recovered without complications and was discharged after seven days.

After 6 months, the patient returned for follow-up without abdominal discomfort, jaundice, nausea, vomiting, or other symptoms, and reexamination of the abdominal USG showed no dilatation of the biliary tree, a smooth pancreas, no dilatation of the pancreatic duct, and no signs of pancreatitis.

## Discussion and conclusion

Most patients with type III CCs complain of abdominal pain, followed by nausea/vomiting, fever, or jaundice [4, 5]. The present case had clinical manifestations and auxiliary examination results that were similar to those of patients with classic CCs, even though the choledochocele did not show a bulge in the duodenum. In addition to the symptoms listed above, to guide treatment, it was essential to understand the anatomy of the distal bile duct prior to surgery and the nature of type III CCs, highlighting that endoscopic sphincterotomy alone is not effective [6–8]. Before the 1990s, oral and intravenous cholangiography were commonly used for choledochoceles diagnosis [9–11]. At present, abdominal USG is a good initial screening method and ERCP is used to carefully define the biliary anatomy, including the APBDJ. While many different diagnostic modalities remain in use, confirmation of type III CCs by ERCP appears to be the most reliable, offering the possibility of use as concomitant treatment. During the course of surgery, the choledochoscopy results confirmed the ERCP image. Thus, through literature review and case practice, we believe that a choledochocele should be evaluated using ERCP as the standard diagnostic test. The amylase concentration in bile also has a certain clinical significance in the diagnosis of choledochoceles.

Type III CCs may be lined with duodenal mucosa, and they are less often associated with malignancy than the other types of CCs [3, 6, 12, 13]. Anatomically accessible type III CCs, in conjunction with lower risk of biliary malignancy, have led to the shift from surgical treatment to endoscopic treatment of choledochoceles [10, 13–15]. Ideally, a treatment’s goal should be to reduce the risk of cancer and maintain a normal flow of pancreatic and biliary secretions.

In the case reported here, the choledochal cyst was small, with a diameter of < 3 cm, the histological classification of the cyst wall was not identified, and the choledochocele did not protrude from the duodenum. Thus, transduodenal excision or sphincteroplasty were not recommended for treatment. We believe that Roux-en-Y choledochojejunostomy could be an effective method for treating type III CCs and choledochus removal would greatly reduce the risk of malignancy by decreasing exposure of the biliary epithelium to toxic pancreatic secretions. It is evident that the differential propensity toward cancer by different types of mucosa has not been adequately studied. Thus, we recommend regular follow-up examinations in patients with choledochoceles.

In summary, Type III choledochal cysts, the rarest choledochal cysts, can only be diagnosed by ERCP, the gold standard. In addition, the amylase concentration in bile has a certain significance for the diagnosis of type III CCs. The case reported here indicates that Roux-en-Y choledochojejunostomy may be the best treatment choice for type III CCs that do not protrude from the duodenum.

## Data Availability

Please contact the corresponding author if you are interested in getting the datasets used in this study.

## Abbreviations

CCs: Choledochal cysts
CBD: Common bile duct
USG: Ultrasonography
WBC: White blood cell
CT: Computed tomography
RR: Reference range
MRCP: Magnetic resonance cholangiopancreatography
APBDJ: Anomalous pancreatobiliary duct junction
ERCP: Endoscopic retrograde cholangiopancreatography
